# *In vitro* evaluation of the antimicrobial activity of antiseptics against clinical *Acinetobacter baumannii* strains isolated from combat wounds

**DOI:** 10.3389/fmicb.2022.932467

**Published:** 2022-10-04

**Authors:** Tetyana Valeriyivna Denysko, Oleksandr Adamovych Nazarchuk, Oleksandr Gruzevskyi, Nataliia Ànatoliivna Bahniuk, Dmytro Valeriiovych Dmytriiev, Roman Mykolayovych Chornopyschuk, Vira Volodymyrivna Bebyk

**Affiliations:** ^1^Department of Microbiology, Virology and Immunology, National Pirogov Memorial Medical University, Vinnytsya, Ukraine; ^2^Department of Microbiology, Virology and Immunology Odessa National Medical University, Odessa, Ukraine; ^3^Department of Anesthesiology, Intensive care, and Emergency Medicine, National Pirogov Memorial Medical University, Vinnytsya, Ukraine; ^4^Department of General Surgery, National Pirogov Memorial Medical University, Vinnytsya, Ukraine

**Keywords:** *Acinetobacter baumannii*, antiseptics, infection, combat wound, resistance, biofilms, antibiofilm-forming activity

## Abstract

Healthcare-associated infections (HCAIs) are among the most prominent medical problems worldwide. In the context of increasing antibiotic resistance globally, the use of antiseptics as the main active agent and potentiator of antibiotics for the treatment of purulent-inflammatory complications of traumatic wounds, burns, and surgical wounds can be considered to tackle opportunistic infections and their prevention during war. This study presents a comparative investigation of the antimicrobial efficacy of antiseptics used for surgical antisepsis and antiseptic treatment of skin, mucous membranes, and wounds against multidrug-resistant clinical isolates of *Acinetobacter baumannii* as a wound pathogen of critical priority (according to the WHO). It was found that strains of *A. baumannii*, which have natural and acquired resistance to antimicrobial drugs, remain susceptible to modern antiseptics. Antiseptic drugs based on decamethoxine, chlorhexidine, octenidine, polyhexanide, and povidone-iodine 10% and 2% provide effective bactericidal activity against *A. baumannii* within the working concentrations of these drugs. Chlorhexidine and decamethoxine can inhibit biofilm formation by *A. baumannii* cells. In terms of bactericidal properties and biofilm formation inhibition, chlorhexidine and decamethoxine are the most effective of all tested antiseptics.

## Introduction

Based on the multicriteria decision analysis technique, WHO experts—together with researchers from the Comprehensive Infectious Disease Center Tübingen (CIDiC) at the University of Tübingen, Germany—published a list of “priority pathogens” – 12 species of bacteria that pose the greatest threat to human health (2017). This list was prepared as a part of the efforts by the WHO to combat the increasing momentum of antimicrobial resistance worldwide. According to the necessity of developing new strategies for resistance mitigation and creating new antibiotics, the bacteria on this list are divided into extremely high priority (critical priority group), high priority, and medium priority. *Acinetobacter*, *Pseudomonas*, and various species of the family *Enterobacteriaceae* (including *E. coli*, *Klebsiella spp.*, *Serratia*, and *Proteus*) were included in the group of critical priority. These bacteria have developed resistance to a wide range of antibiotics, including carbapenems and third-generation of cephalosporins, which are considered reserve antibiotics among the available antibiotics for the treatment of multidrug-resistant bacterial infections (World Health Organization [WHO], 2017; Centers for Disease Control and Prevention [CDC], 2019; Talebi Bezmin Abadi et al., 2019).

Carbapenem-resistant *A. baumannii* was classified as the most serious menace in the “critical group” of priority. This non-fermenting gram-negative bacterium is a major cause of severe infections associated with medical care, such as pneumonia associated with pulmonary ventilation (artificial lung ventilation), bloodstream infections, bacteremia, urinary tract infections, and wound infections, especially in patients with burns and postoperative pains (Gheorghe et al., 2021; Sloczynska et al., 2021). During war, the impact of *A. baumannii* on the development of combat wound infections and other infections has been reported previously (Calhoun et al., 2008).

In this context, with increasing resistance of *A. baumannii* toward reserve antibiotics, such as carbapenems and polymyxins, therapeutic possibilities are very limited or absent in some cases of infections caused by bacteria with pan-antimicrobial resistance. This situation encourages researchers worldwide to seek and study new therapeutic strategies as an alternative to antibiotics (Fournier et al., 2006; Kempf and Rolain, 2012; Thomas et al., 2019; Ibrahim et al., 2021; Lin et al., 2021; Park et al., 2021; Pimentel et al., 2021; Shahid et al., 2021; Sloczynska et al., 2021).

In addition, a significant correlation between multidrug resistance and biofilm formation of *A. baumannii* clinical isolates was established (Han et al., 2014; Kim et al., 2015; Dafopoulou et al., 2016; de Campos et al., 2016; Duarte et al., 2016; Qi et al., 2016; Zhang et al., 2016; Bardbari et al., 2017). Developing biofilm-specific countermeasures is a step toward limiting and containing biofilm-associated infections (Han et al., 2014; Kim et al., 2015; Dafopoulou et al., 2016; de Campos et al., 2016; Duarte et al., 2016; Qi et al., 2016; Zhang et al., 2016; Bardbari et al., 2017).

Effective management of bacterial bioload is an important element of wound care, as any injury is characterized by a high probability of infection. The use of antiseptics to combat colonization and infection directly at the portal of entry is a vital step in preventing further infectious complications. Antiseptics are defined as antimicrobial substances that are non-damaging to living tissue/skin while reducing the possibility of infection, sepsis, or putrefaction. Among the variety of antiseptic preparations, ñationic surfactants (CSs) and complex compounds of iodine and the synthetic polymer polyvinylpyrolidone (povidone-iodine) deserve special attention. Because of their important properties, such as the non-selectivity of the antimicrobial effect on the bacterial cell, sufficient biological safety, lack of toxic effect, and weak irritating effect on the skin, mucous membranes, and tissues of wound surfaces, CSs are considered the most promising (Junka et al., 2014; Alvarez-Marin et al., 2017; Davis et al., 2017; Hoekstra et al., 2017; Santos et al., 2017; Shepherd et al., 2018; Htun et al., 2019; Jeronimo et al., 2020; Huang et al., 2021; Krasowski et al., 2021; Lin et al., 2021; Loose et al., 2021).

This work studied the antimicrobial activity of antiseptics against planktonic bacterial forms and biofilm formation of multidrug-resistant clinical strains of *A. baumannii* isolated from combat wounds with infectious and inflammatory complications.

## Materials and methods

### Bacterial strains

The activity of antiseptics was determined against clinical antibiotic-resistant strains of *A. baumannii* (*n* = 42). Clinical strains were isolated from patients with infectious complications from combat burn wounds of different localizations received during the war conflict in Ukraine. A strain from the American Type Culture Collection (ATCC) of *A. baumannii BAA-747* was used as a control. All clinical isolates were identified by standard microbiological methods, considering their morphological, tinctorial, cultural, and biochemical properties. To study the biochemical profile, «NEFERM test 24» («Erba Lachema») was used.

Forty-two isolates (74% of the total) were identified as multidrug-resistant strains according to the definition criteria MDR, XDR, and PDR in *Acinetobacter spp*., proposed by the European Centre for Disease Prevention and Control (ECDC) and the Centers for Disease Control and Prevention (CDC). Multidrug-resistant (MDR) was defined as acquired non-susceptibility to at least one agent in three or more antimicrobial categories per the guidelines.

After the antimicrobial susceptibility testing—which was performed using the disc diffusion method according to the Clinical Laboratory Standards Institute (CLSI), the EUCAST European Committee on Antimicrobial Susceptibility Testing (EUCAST) standards—there were found phenotypic antimicrobial resistance of clinical strains of *A. baumannii* to antibiotics belonging to the antimicrobial categories of aminoglycosides, carbapenems, antipseudomonal and extended-spectrum cephalosporins, antipseudomonal penicillins + β-lactamase inhibitors, antipseudomonal fluoroquinolones, antipseudomonal tetracyclines; we found the resistance of studied isolates to tobramycin (52.38%), gentamicin (59.52%), amikacin (78.57%), imipenem (57.14%), meropenem (64.29%), ceftazidime (92.34%), to cefoperazone-sulbactam (73,8%), to cefepime (95.24%), piperacillin-tazobactam (80.95%), to ciprofloxacin (88.1%), to levofloxacin (83.33%), and also to ampicillin-sulbactam (38.1%), to doxycycline (33.33%). Additionally, these isolates were susceptible to colistin.

### Antiseptics

The sensitivity of the reference and clinical strains of *A. baumannii* was studied for the following quaternary ammonium antiseptics in their working concentrations: 0.1% decamethoxine ((1,10-decamethylene bis (N, N-dimethylmethoxycarbonylmethyl) ammonium dichloride) and a drug based on it – 0.02% decamethoxine, 0.05% chlorhexidine ((1E)-2-[6-[amino-[(E)-[amino-(4-chloroanilino)methylidene] amino]methylidene]amino] hexyl]-1-[amino-(4-chloroanilino)methylidene] guanidine), 0.1% octenidine (N-octyl-1-[10-(4-octyliminopyridin-1-yl)decyl]pyridin-4-imine), 0.1% polyhexanide (1-(diaminomethylidene)-2-hexylguanidine), and 0.01% miramistin (benzyl-dimethyl-[3-(tetradecanoylamino) propyl]azanium; chloride). Also, an antiseptic from the group of halogenated compounds—povidone-iodine (1-ethenylpyrrolidin-2-one; molecular iodine) with an initial concentration of 10% and recommended working dilutions of 1:5 (2%) and 1:10 (1%) was used in the study.

Well-known antiseptic substances were used in the form of pharmaceutical products available in Ukraine: povidone-iodine (PVP-I, Betadine^®^, EGIS Pharmaceuticals PLC, Hungary), octenidine dihydrochloride [Octenisept^®^ farblos/incolore, Schulke & Mayr GmbH, Germany], polyhexanide solution [Prontosan^®^, B Braun Medical, Germany], decamethoxine 0.1% (was prepared from the substance powder of Decamethoxine^®^, Yuria-Pharm, Ukraine), decamethoxine 0.02% (Decasan^®^, Yuria-Pharm, Ukraine) chlorhexidine digluconate (Chlorhexidine-Viola^®^ Viola, FF, JSC, Ukraine), ìiramistin 0.01% (Miramistin^®^, Darnitsa PrAT, Ukraine).

### Susceptibility assays on planktonic cells

#### Minimum inhibitory concentration and minimum bactericidal concentration determination

The study evaluated the antimicrobial activity of antiseptics by determining their minimum inhibitory (bacteriostatic) and bactericidal concentrations (MIC and MBC, respectively) against reference and clinical strains of *A. baumannii*. The standard macro method of double serial dilutions, according to guidelines of Ukraine No167 dated April 5, 2007, and Standards for Antimicrobial Susceptibility Testing, in accordance with the Clinical and Laboratory Standards Institute guidelines (CLSI, USA), was used (Ministry of Health of Ukraine, 2007; Clinical and Laboratory Standards Institute [CLSI], 2014). Microorganisms were cultured using Mueller-Hinton broth (HiMedia Laboratories, India). Successive two-fold dilutions of antiseptics were prepared to start from working concentrations. For inoculation, a microbial suspension corresponding to 0.5 McFarland’s standard was used, diluted 100 times in nutrient broth, after which the concentration was approximately 5 × 10^6^ CFU/ml. MIC was registered 24 h after incubation at 37°C as the lowest concentration of the drug that prevents visible growth. For determining MBC, samples within the turbidity threshold after 24 h were plated on nutrient agar, evaluating their growth after another 24 h.

#### Bacteriostatic index of antiseptic activity and bactericidal index of antiseptic activity determination

Additionally, a comparative analysis of the antimicrobial efficacy of antiseptics by the index of antiseptic activity (IAA) was performed, differentiating bacteriostatic and bactericidal effects according to the method (Krasilnikov, 1995; Andreeva et al., 2018): bacteriostatic index of antiseptic activity (BS IAA) was calculated as the ratio of the working concentration of the antiseptic to its minimum inhibitory concentration relative to the pathogen, and the bactericidal index of antiseptic activity (BC IAA) was calculated as the ratio of the working concentration of the antiseptic to its minimum bactericidal concentration. IAA is an indicator that allows comparison of the antiseptic activity of drugs regardless of their working concentration. According to the method, the antiseptic was evaluated as active by IAA > 4 because, under natural conditions, the activity of antiseptics is reduced by an average of 4 times.

### Susceptibility assays on biofilm formation

#### Quantitative crystal violet assay

The biofilm-forming ability was determined by the microtiter-plate Christensen test (quantitative crystal violet assay). The effect of antiseptics on biofilm formation (on immature biofilms) was assessed by reproducing biofilms with the addition (simultaneously with bacterial culture) of antiseptics at sub-inhibitory concentrations for 24 h and subsequent spectrophotometric ODU (optical density units) assessment. Each of the 42 strains of *A. baumannii* and each antiseptic corresponded to its own sub-inhibitory concentration, which represented one-third of the MIC and, on average, it was 6.44 ± 1.19 μg/ml for decamethoxine, 18.41 ± 3.27 μg/ml for chlorhexidine, 5.79 ± 0.76 μg/ml for octenidine, 12.61 ± 1.4 μg/ml for polyhexanide, 12.07 ± 1.12 μg/ml for miramistin, and 838.68 ± 84.01 μg/ml for povidone-iodine. Each strain was exposed to a specific sub-inhibitory concentration for it.

Briefly, bacteria isolated from fresh agar plates were inoculated into a tube filled with sterile tryptic soy broth (TSB, EMD Millipore, USA) with 1% glucose and incubated at 37°C for 24 h. This culture was diluted 1:100 into the fresh media. Then, 200 μL of the suspension was added to a sterile 96-well flat-bottom microtiter plate (USA Scientific, Inc). Sub-bacteriostatic concentrations of antiseptics were added to the wells with the medium and bacterial cells (test agent at 1/3xMIC). The bacterial suspension of each well was carefully removed and washed three times with phosphate buffer saline, pH 7.2 (Sigma, USA; cat. no. P-3813). Then, the bacteria were fixed with absolute methanol and stained with 220 μl of crystal violet 0.1% w/v (Merck, Germany) for 15 min at room temperature. Each well was washed three times with PBS to remove unbound CV dye. After drying, 220 μL of ethanol (95%) was added to each well. All spectrophotometric measurements were performed on a STAT FAX^®^4300 spectrophotometer (Netherlands) at a wavelength of 620 nm. The experiment was carried out in triplicate, separately for each strain, and the average value was calculated. The cut-off optical density (ODc) was indicative of biofilm formation and was defined as the sum of the arithmetic mean of negative controls and a triple value of its standard deviation (ODc = $\ddot{x}$ + 3σ). A TSB without bacterial suspension incubated in the microtiter plate was used as a negative control. Interpretation of the results was carried out according to the conventional methodology. Thus, the ability of microorganisms to form biofilms was assessed as low at optical density < 0.120, average – at optical density = 0.121–0.239, and high at optical density > 0.240. The optical density for each isolate without the use of antiseptics was taken as a control against which the results were compared (Christensen et al., 1985; Diriba et al., 2020).

### Statistical analysis

To assess the degree of reliability of the obtained results, we used the variation-statistical method of analysis, calculating the arithmetic mean (M), the arithmetic mean error (m), the mean error (t), and the reliability of the difference (p). Statistical processing was performed using a Microsoft Office Excel spreadsheet (V. 16.0.5056.1000, 2016) and Statistica software packages (v. 12.5.192.7, StatSoft Inc.). The differences were considered statistically significant at *p* ≤ 0.05 and insignificant at *p* > 0.10. To establish the relationship between susceptibility to antiseptics and biofilm formation of the studied microorganisms, we used Pearson’s Correlation Coefficient (r) to determine the absolute value that characterizes the relationship level between variables.

## Results

### Antimicrobial activity on planktonic *Acinetobacter baumannii* cells

The study found a high *in vitro* efficacy of the antiseptics evaluated here against *A. baumannii.* The quantitative bacteriostatic and bactericidal actions of the studied antiseptics are presented in Table 1. Coefficients of reliability of the difference between the minimum inhibitory concentrations of the studied antiseptics are shown in Table 2. The coefficients of reliability of the difference between the minimum bactericidal concentrations are presented in Table 3.

**TABLE 1:** Characteristics of susceptibility to antiseptics of clinical strains of *A. baumannii* isolated from patients with microbial complications of combat wounds, in μg/ml (arithmetic mean ± arithmetic mean error: M ± m).

Antiseptics	*A. baumannii* (*n* = 42)			
	MIC *	*p*_1_***	MBC **	**p*_2_* ^†^
Decamethoxine 0.1%	18.8 ± 3.78	–	36.17 ± 5.17	–
Decamethoxine 0.02%	19.83 ± 3.35	> 0.05	38.32 ± 6.34	> 0.05
Miramistin 0.01%	36.22 ± 3.37	<0.001	67.95 ± 5.03	<0.001
Chlorhexidine 0.05%	55.24 ± 9.81	<0.001	123.14 ± 23.15	<0.001
Octenidine 0.1%	17.8 ± 2.27	> 0.05	36.82 ± 4.69	> 0.05
Polyhexanide 0.1%	37.82 ± 4.19	<0.01	72.37 ± 7.94	<0.001
Povidone-Iodine 10.0%	2516.03 ± 252.02	–	3910.26 ± 416.28	–

*MIC: minimum inhibitory concentration; **MBC: minimum bactericidal concentration, ***p_1_: coefficients of reliability of the difference between the minimum inhibitory concentrations of the studied antiseptics in comparison with decamethoxine 0.1%; ^†^*p*_2_, coefficients of reliability of the difference between the minimum bactericidal concentrations of the studied antiseptics in comparison with decamethoxine 0.1%.

**TABLE 2:** Coefficients of reliability of the difference between the minimum inhibitory concentrations of the studied antiseptics against clinical strains of *A. baumannii* (p_1_).

	MIC* of Decamethoxine 0.1%	MIC* of Decamethoxine 0.02%	MI*C of Chlorhexidine 0,05%	MIC* of Octenidine 0.1%	MIC* of Miramistin 0.01%	MIC* of Polyhexanide 0.1%
MIC* of Decamethoxine 0.1%	1.0000	> 0.10	0.001	> 0.10	<0.001	<0.01
MIC* of Decamethoxine 0.02%	> 0.10	1.0000	0.001	> 0.10	<0.001	<0.01
MIC* of Chlorhexidine 0.05%	0.001	0.001	1.0000	<0.001	> 0.05	>0.10
MIC* of Octenidine 0.1%	> 0.10	>0.10	<0.001	1.0000	<0.001	<0.001
MIC* of Miramistin 0.01%	<0.001	<0.001	> 0.05	<0.001	1.0000	> 0.10
MIC* of Polyhexanide 0.1%	<0.01	<0.01	> 0.05	<0.001	> 0.10	1.0000

*MIC, minimum inhibitory concentration of antiseptics.

**TABLE 3:** Coefficients of reliability of the difference between the minimum bactericidal concentrations of the studied antiseptics against clinical strains of *A. baumannii* (p_2_).

	MBC* of Decamethoxine 0.1%	MBC* of Decamethoxine 0.02%	MBC* of Chlorhexidine 0.1%	MBC* of Octenidine 0.1%	MBC* of Miramistin 0.01%	MBC* of Polyhexanide 0.1%
MBC* of Decamethoxine 0.1%	1.0000	> 0.10	<0.001	> 0.10	<0.001	<0.001
MBC* of Decamethoxine 0.02%	> 0.10	1.0000	<0.001	> 0.10	<0.001	<0.01
MBC* of Chlorhexidine 0.1%	<0.001	<0.001	1.0000	<0.001	< 0.05	> 0.10
MBC* of Octenidine 0.1%	> 0.10	>0.10	<0.001	1.0000	<0.001	<0.001
MBC* of Miramistin 0.01%	<0.001	<0.001	< 0.05	<0.001	1.0000	> 0.10
MBC* of Polyhexanide 0.1%	<0.001	< 0.05	> 0.10	<0.001	> 0.10	1.0000

*MBC, minimum bactericidal concentration.

The highest activity against clinical strains of *A. baumannii* among the studied antiseptics was found in decamethoxine (0.1% and 0.02%) and octenidine (0.1%), as evidenced by their bacteriostatic and bactericidal concentrations: the average values of MIC were 18.8 ± 3.78 μg/ml; 19.83 ± 3.35 μg/ml and 17.38 ± 2.27 μg/ml, respectively; MBC values were 36.17 ± 5.17 μg/ml; 38.32 ± 6.34 μg/ml and 36.82 ± 4.69 μg/ml, respectively (Table 1).

Miramistin and polyhexanide demonstrated fairly high bacteriostatic and bactericidal activity against strains of *A. baumannii*. Effective inhibition of *A. baumannii* growth was observed with miramistin (36.22 ± 3.37 μg/ml) and polyhexanide (37.82 ± 4.19 μg/ml). There were determined bactericidal effects of miramistin (67.95 ± 5.03 μg/ml) and polyhexanide (72.37 ± 7.94 μg/ml).

Among the quaternary ammonium antiseptics studied, clinical isolates of *A. baumannii* were the least susceptible to chlorhexidine. The average values of bacteriostatic concentrations of chlorhexidine were 55.24 ± 9.81 μg/ml, and bactericidal properties were determined in the presence of concentrations that were 2.23 times higher and amounted to 123.14 ± 23.15 μg/ml (Table 1).

The bactericidal effect of decamethoxine on *A. baumannii* exceeded that of chlorhexidine by 3.21 times, and this difference had a high statistical value (*p* < 0.001). Decamethoxine showed biocidal properties that significantly exceeded the bactericidal actions of miramistin 1.77 times (*p* < 0.001) and polyhexanide 1.89 times (*p* < 0.01). Similar properties were shown by octenidine with reliable values of the difference in results. The bacteriostatic and bactericidal concentrations of miramistin and polyhexanide were similar in their efficacy [the coefficients of reliability of the difference between the results were not significant (*p* > 0.10)]. The miramistin MBC was 1.81 times lower (*p* < 0.05) than the chlorhexidine MBC (Tables 2, 3).

Bacteriostatic concentrations of povidone-iodine against *A. baumannii* averaged 2516.03 ± 252.02 μg/ml, and bactericidal concentrations were 3910.26 ± 416.28 μg/ml (Table 1). As povidone-iodine belongs to another chemical group of antiseptics (halide-containing compounds), in contrast to other studied drugs (cationic surfactants), its active substance is present in the initial solution in much higher concentrations, and it is impossible to compare these drugs.

**FIGURE 1:**
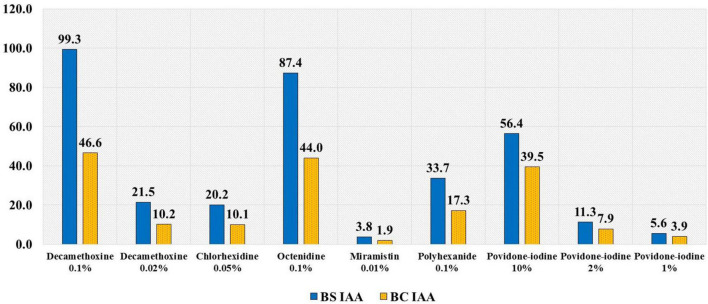
The average values of bacteriostatic (BS) and bactericidal (BC) IAA in relation to clinical isolates of *A. baumannii.*

Comparative analysis of antiseptics was also performed using a differentiated indicator of IAA, calculating BS IAA and BC IAA, which allowed us to assess the feasibility of using certain concentrations of active substances in the initial working solution of the drug. As povidone-iodine dilutions of 1:5 and 1:10 are recommended, such concentrations (2% and 1%) were also included in the comparative analysis as stock solutions of the drug (Figure 1).

The bacteriostatic index of antiseptic activity (BS IAA) of decamethoxine 0.1% was 99.3, and the bactericidal index of antiseptic activity (BC IAA) for decamethoxine 0.1% was 46.6. The BS IAA and BC IAA for decamethoxine 0.02% were 21.5 and 10.2, respectively. For chlorhexidine 0.05%, BS IAA and BC IAA values of 20.2 and 10.1 were determined. The BS IAA and BC IAA values for octenidine 0.1% were 87.4 and 44.0, respectively. For miramistin, 0.01%, the values of BS IAA and BC IAA were 3.8 and 1.9; for polyhexanide, 0.1%, and BS IAA and BC IAA values of 33.7 and 17.3 were determined. The values of BS IAA and BC IAA for povidone-iodine 10% were 56.4 and 39.5; for povidone-iodine 2%, 11.3 and 7.9; for povidone-iodine 1%, 5.6 and 3.9, respectively.

### *In vitro* effect of antiseptics on the biofilm-forming activity of *Acinetobacter baumannii*

All the tested strains were efficient in forming biofilms. The multidrug-resistant clinical isolates of *A. baumannii* were found to have medium biofilm-forming properties. The average value of the degree of dye absorption by biofilms in the control wells was 0.230 ± 0.05 optical density units (ODU).

This study showed that sub-bacteriostatic concentrations of decamethoxine (an average of 6.44 ± 1.19 μg/ml) and chlorhexidine (an average of 18.41 ± 3.27 μg/ml) reliably (*p* < 0.001) inhibited biofilm formation by *A. baumannii* for 24 h. Under the effect of decamethoxine, the average value of the optical density of *A. baumannii* biofilms decreased 1.11 times compared with the control. It amounted to 0.207 ± 0.01 ODU, and in the presence of chlorhexidine, it was increased by 1.15 times and was 0.200 ± 0.01 ODU.

**FIGURE 2:**
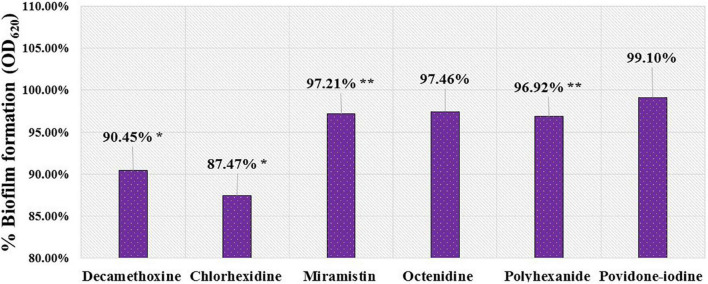
Percentage indicator of the biofilm-forming ability of *A.baumannii* (*n* = 42) in the presence of sub-inhibitory concentrations of antiseptics compared to the untreated control. *- coefficient of reliability (*p* < 0.001); **- coefficient of reliability *p* < 0.01.

Sub-inhibitory concentrations of octenidine (an average of 5.79 ± 0.76 μg/ml), polyhexanide (an average of 12.61 ± 1.4 μg/ml), miramistin (an average of 12.07 ± 1.12 μg/ml), and povidone-iodine (an average of 838.68 ± 84.01 μg/ml) showed a lower anti-biofilm-forming effect, and the difference was not statistically significant. Reliability coefficients ranged from *p* = 0.29 to *p* = 0.93. The results of these experiments are summarized in percentages of the biofilm-forming ability of *A. baumannii* isolates in the presence of antiseptics compared to the untreated control (Figure 2).

On evaluating the anti-biofilm effects of the said antiseptics, decamethoxine and chlorhexidine exhibited the most pronounced effects on immature biofilms. The inhibitory effect was 90.45% and 87.47% compared to the control (100%). For miramistin, octenidine, polyhexanide, and povidone-iodine, these values were 97.21%, 97.46%, 96.92%, and 99.10%, respectively (Figure 2). Decamethoxine and chlorhexidine in sub-bacteriostatic concentrations showed the most pronounced effects on immature biofilm. They significantly inhibited biofilm formation by *A. baumannii* by 9.55% (*p* < 0.001) and 12.53% (*p* < 0.001), respectively, compared to the control. Miramistin, octenidine, polyhexanide, and povidone-iodine showed a less pronounced effect. Miramistin inhibited biofilm formation by 2.79% (*p* < 0.01); octenidine, by 2.54% (*p* > 0.10); polyhexanide, by 3.08% (*p* < 0.01); and povidone-iodine, by 0.9% (*p* > 0.10), comparably to the untreated control.

A negative correlation was found between the biofilm-forming properties of strains in the presence of sub-bacteriostatic concentrations of the studied antiseptics and their susceptibility to them. The Pearson correlation coefficients for decamethoxine, miramistin, octenidine, polyhexanide, and povidone-iodine were *r* = –0.76, –0.92, –0.59, –0.87, –0.9, respectively. Thus, the inhibition of biofilm-forming properties depends on the concentration of the antiseptic and not on the susceptibility of *A. baumannii* to these antiseptics.

The biofilm-forming properties of *A. baumannii* clinical strains did not correlate well with their susceptibility to chlorhexidine. This was indicated by a low r-Pearson coefficient (*r* = 0.15).

## Discussion

Healthcare-associated infections (HCAIs) are among the most prominent medical problems worldwide. The US Center for Disease Control and Prevention identifies that nearly 1.7 million hospitalized patients annually acquire HCAIs while being treated for other health issues and that more than 98,000 patients (one in 17) die due to these (Hoekstra et al., 2017).

The rapidly evolving nature of *A. baumannii* multidrug-resistant strains is a cause of concern. It accounts for approximately 2–10% of all gram-negative hospital-acquired infections in intensive care units (Calhoun et al., 2008). In 2017, carbapenem-resistant *Acinetobacter* caused an estimated 8,500 infections in hospitalized patients and 700 estimated deaths in the USA (Centers for Disease Control and Prevention [CDC], 2019).

In the context of a global increase in antibiotic resistance, the use of antiseptics as the main active agent and potentiator of antibiotics is of great importance for treating patients with infected post-traumatic and surgical wounds and burns (Kempf and Rolain, 2012; Thomas et al., 2019; Lin et al., 2021; Park et al., 2021). In this study, the inhibitory and bactericidal effects of the selected antiseptics on *A. baumannii* planktonic and biofilm-forming states were evaluated.

Maillard et al. (2021) reviewed previous studies of susceptibility to modern antiseptics and reported decreased biocide susceptibility for all biocides. So far, planktonic forms of *A. baumannii* have shown only a weak adaptive or no response to chlorhexidine, octenidine, polyhexanide, and povidone-iodine. The MIC and MBC values of antiseptics are known to differ, increasing in the presence of multidrug-resistant isolates. In the case of *A. baumannii*, such a probability of variability has prompted us to research strains isolated from combat burn wounds from different localizations. Our study found a fairly high *in vitro* efficacy of the tested antiseptics, which are commonly used.

The MIC and MBC values of quaternary ammonium compound (QAC) antiseptics, such as decamethoxine and octenidine, showed that they were the most effective drugs. For QAC and halogen compound antiseptics, as their IAA (BS and BC) was >4, they were considered active. The ratio of BC to BS IAAs varied between 0.47 and 0.5 for QAC antiseptics. For different concentrations of povidone-iodine, this ratio was 0.70, demonstrating that it had the highest bactericidal effect.

Index of antiseptic activity (IAA) values were highest (IAA > 4) for the antiseptics decamethoxine (0.1%), octenidine (0.1%), and povidone-iodine (10%), which correlated with high concentrations of active substances in the initial working solutions of these drugs. On the other hand, 0.02% decamethoxine, 0.05% chlorhexidine, and 0.1% polyhexanide exhibited lower IAA values; however, their BS and BC IAA values exceeded the threshold (4) by 5.05–7.4 times and 2.53–4.0 times, respectively; these concentrations were effective against multidrug-resistant *A. baumannii*. On the contrary, the effectiveness of 0.01% miramistin against multidrug-resistant *A. baumannii* was found insufficient (BS IAA = 3.8; BC IAA = 1.9, both < 4). The feasibility of using 1.0% povidone-iodine is questionable as the BS IAA (=5.6) is above the threshold value, while the BC IAA (=3.9) is not. In order to avoid creating selective conditions for the emergence of resistant strains, it may be appropriate to use concentrations of povidone-iodine of no less than 2%.

The results of the current study are largely consistent with those from other countries. Many researchers have reported a decrease in the susceptibility of many microorganisms to chlorhexidine (Saleem et al., 2016; Maya et al., 2021; Leshem et al., 2022). Leshem et al. (2022) demonstrated reduced susceptibility of gram-negative bacteria, including *A. baumannii*, to chlorhexidine but, at the same time, emphasized that none of these isolates appeared to be chlorhexidine-tolerant based on their MIC values. Another study showed the effectiveness of chlorhexidine and povidone-iodine against 81 *A. baumannii* isolates. However, 18.51% of the isolates were resistant to one-third of diluted povidone-iodine (Lanjri et al., 2017). A study by López-Rojas et al. (2017) highlighted that polyhexanide demonstrated bactericidal activity against all high-risk clones of multidrug-resistant nosocomial pathogens (including *A. baumannii*) at significantly lower concentrations and times of activity than those commercially used. The usefulness of octenidine for eradicating emergent, highly resistant Gram-negative nosocomial pathogens has been previously reported (Alvarez-Marin et al., 2017).

Increased tolerance of biofilms toward antimicrobials and their involvement in recurring infections has prompted the development of anti-biofilm strategies (Maillard et al., 2021). Therefore, the second part of this study aimed to evaluate the ability of commercially available antiseptics to prevent biofilm formation. QAC antiseptics and the halogenated compound antiseptic, povidone-iodine, exhibited different effects on the formation stage of *A. baumannii* biofilms. Previously, chlorhexidine and octenidine were found to possess the greatest efficacy against mature biofilms of clinical multidrug-resistant microorganisms, including *A. baumannii* (Günther et al., 2021). We investigated the effect of antiseptics on immature biofilms to prevent their formation. Our study also confirms that chlorhexidine had the highest antibiofilm activity against clinical isolates.

In the presence of sub-bacteriostatic chlorhexidine concentrations, our clinical strains’ biofilm-forming properties negatively correlated with their susceptibility to this antiseptic (r = 0.15). Owing to the significant inhibition of biofilm formation and the positive correlation of this property with the susceptibility of *A. baumannii* isolates toward this antiseptic, chlorhexidine should be considered an effective agent. Sub-bacteriostatic concentrations of decamethoxine significantly inhibited biofilm formation. This property was inversely correlated with the decamethoxine susceptibility of the strains (r = –0.76). Thus, the biofilm-forming activity of *A. baumannii* strains was inversely proportional depending on the concentration of the said antiseptic used. Analyzing the obtained results, we can see that bacteriostatic and higher concentrations of decamethoxine can provide reliable protection against biofilms.

Sub-bacteriostatic concentrations of octenidine, polyhexanide, miramistin, and povidone-iodine showed a low anti-biofilm-forming effect in *A. baumannii*, and the difference relative to the control was not statistically significant. A strong negative correlation between the biofilm-forming properties of *A. baumannii* in the presence of sub-bacteriostatic concentrations of these mentioned antiseptics and their susceptibility toward them indicates the dependence of these properties on antiseptic concentration, not on the increased susceptibility of individual strains. The correctness of our interpretation is confirmed by the studies of Loose et al. (2021) where octenidine and polyhexanide prevented biofilm formation in Escherichia coli, Pseudomonas aeruginosa, and Proteus mirabilis, depending on antiseptic concentration (Loose et al., 2021). Moreover, 0.25% (w/w) of povidone-iodine completely eradicated the biofilms of all multidrug-resistant microorganisms (Capriotti et al., 2018). It follows that the use of these QAC antiseptics in concentrations lower than MIC may stimulate the protective mechanisms of bacteria, as well as biofilm formation, which needs further research.

According to previous literature (Qi et al., 2016), a higher risk of biofilm formation is closely associated with multi-resistant strains of *A. baumannii* than with susceptible ones since the ability to form a biofilm is an important factor of resistance. At the same time, the relationship between the biofilm formation of antibiotic-resistant clinical strains of bacteria and their susceptibility to antiseptics has not yet been clearly established (Babapour et al., 2016; Bardbari et al., 2017; Guo and Xiang, 2017; Hall and Mah, 2017; Haque et al., 2018). Our research found a similar pattern for clinical strains of *A. baumannii*. A statistically negative correlation was found between the susceptibility to antiseptics of studied antibiotic-resistant clinical strains and their biofilm-forming capacity in the presence of sub-MIC levels of the studied antiseptics (except for chlorhexidine).

Previous research from different countries and time periods has also dealt with the problem of wound infection, its causative agents, and the effectiveness of modern antiseptics against leading wound pathogens. Different approaches and methods were used to achieve these goals (Koburger et al., 2010; Shen et al., 2011; Abdeyazdan et al., 2014; Amalaradjou and Venkitanarayanan, 2014; Kovalchuk et al., 2014; Günther et al., 2015; Lefebvre et al., 2016; Bukhary and Balto, 2017; Gunther et al., 2017; Hoekstra et al., 2017; Kittinger et al., 2017; Johani et al., 2018; Machuca et al., 2019; Krasowski et al., 2021). An important task for us is to monitor wound pathogens in our country during wartime and analyze the effectiveness of available antiseptics against them. Given the increasingly global spread of antibiotic resistance, the susceptibility of various microorganisms to biocides may constantly change. Thus, a comprehensive study of the antimicrobial action of antiseptics, a detailed analysis of the obtained data, and a study of the experience of researchers from different countries are necessary to fight against infectious complications of wounds and burns.

## Conclusion

Modern surfactant-active antiseptics include decamethoxine, chlorhexidine, octenidine, polyhexanide, and halogenated compound povidone-iodine (10 and 2%), which provide effective antimicrobial activity against planktonic multidrug-resistant *A. baumannii* clinical strains colonizing combat wounds and burns, while miramistin (0.01%) was not effective. Sub-bacteriostatic concentrations of decamethoxine and chlorhexidine reliably inhibited the formation of biofilms by clinical strains of *A. baumannii* that colonized combat wounds (*p* < 0.001). The high antibiofilm-forming properties of chlorhexidine that are positively correlated with the susceptibility of *A. baumannii* to this antiseptic demonstrate its effectiveness against biofilm formation. In terms of both bactericidal properties and inhibition of biofilm formation, chlorhexidine and decamethoxine are the most effective of all tested antiseptics.

## Data availability statement

The datasets presented in this study can be found in online repositories. The names of the repository/repositories and accession number(s) can be found in the article/supplementary material.

## Author contributions

TD, NB, DD, and VB performed the antibacterial activity determination and were involved in the review of the literature. ON and RC were involved in collecting microbial cultures. TD, ON, OG, and RC designed the study, wrote the manuscript, participated in the data analysis, and provided critical manuscript revisions for valuable intellectual content. All authors contributed to the interpretation of the results, the revision of the draft manuscript, and the approval of the final version.
